# Supplementary material for: Inorganic-organic competitive coating strategy derived uniform hollow gradient-structured ferroferric oxide-carbon nanospheres for ultra-fast and long-term lithium-ion battery
Source: Nat Commun. 2021 May 20;12:2973. doi: 10.1038/s41467-021-23150-8 (PMC8137936; doi:10.1038/s41467-021-23150-8)
Supplement: Supplementary file 1 — Supplementary Information [file 41467_2021_23150_MOESM1_ESM.pdf]

Supplementary Materials

Inorganic-organic competitive coating strategy derived uniform hollow gradient-structured ferroferric oxide-carbon nanospheres for ultra-fast and long-term lithium-ion battery

Yuan Xia[1], Tiancong Zhao[1], Xiaohang Zhu[1], Yujuan Zhao[1], Haili He[1], Chin-te Hung[1], Xingmiao Zhang[1], Yan Chen[1], Xinlei Tang[1], Jinxiu Wang[1], Wei Li[1]*, and Dongyuan Zhao[1,]*

1. Department of Chemistry and Shanghai Key Lab of Molecular Catalysis and Innovative Materials, Laboratory of Advanced Materials, Fudan University, Shanghai 200433, P. R. China.

E-mail: dyzhao@fudan.edu.cn; weilichem@fudan.edu.cn

This file includes:

Supplementary Figure 1. Morphology characterization of the pure colloidal SiO_2 cores and as-made gradient-structured nanospheres (SiO_2@G-Fe_3O_4@C) after inorganic-organic competitive coating strategy at 210 °C under solvothermal condition.

Supplementary Figure 2. HRTEM images of a broken hollow gradient-structured Fe_3O_4@C (HG-Fe_3O_4@C) nanosphere after mechanical milling for 1 h.

Supplementary Figure 3. TEM image of the hollow carbon nanospheres obtained by etching the hollow gradient-structured Fe_3O_4@C pheres with 0.1 M HF solution to remove of all Fe_3O_4 nanoparticles.

Supplementary Figure 4. TEM images of the hollow hybrid Fe_3O_4/C (HH-Fe_3O_4/C) nanospheres obtained by etching the as-made SiO_2@G-Fe_3O_4@C nanospheres with 1

M NaOH before annealing to remove the outer amorphous carbonaceous layers and colloidal SiO_2 nanosphere cores.

Supplementary Figure 5. Superficial characteristics of the hollow gradient-structured Fe_3O_4@C nanospheres.

Supplementary Figure 6. The TGA curve of the sample after etching by HF for the removal of Fe_3O_4.

Supplementary Figure 7. The XRD pattern of the red product after TG analysis process.

Supplementary Figure 8. TEM images and XRD patterns of the products prepared by different reaction temperature through the inorganic-organic competitive coating strategy.

Supplementary Figure 9. TEM images and thickness curve of time-dependent experiment under the solvothermal condition of 210 °C.

Supplementary Figure 10. TEM images and thickness curve of time-dependent experiment under the solvothermal condition of 180 °C.

Supplementary Figure 11. TEM images of the as-made gradient-structured nanospheres (SiO_2@G-Fe_3O_4@C) with a controllable particle size from 150 to 500 nm and their derived hollow gradient-structured Fe_3O_4@C nanospheres.

Supplementary Figure 12. TEM images of the as-made gradient-structured nanospheres (SiO_2@G-Fe_3O_4@C) with varied shell thicknesses from 20 to 80 nm and their derived hollow gradient-structured Fe_3O_4@C nanospheres.

Supplementary Figure 13. Morphology characterization of the strawberry-like Fe_3O_4@C (S-Fe_3O_4@C) shells coating on the excessive colloidal SiO_2 cores.

Supplementary Figure 14. TEM images of the strawberry-like Fe_3O_4@C (S-Fe_3O_4@C) shells coating on carbon substrate cores with excessive interface.

Supplementary Figure 15. TEM images of the functional silica nanosphere cores coated by the gradient-structured Fe_3O_4@C shell and their derived yolk-shell structured nanospheres.

Supplementary Figure 16. Characterization of the TiO_2@C (G-TiO_2@C) shell coating on the colloidal SiO_2 cores by using titanocene as the reactant through the inorganic-organic competitive coating strategy.

Supplementary Figure 17. Characterization of the NiO@C (G-NiO@C) shell coating on the colloidal SiO_2 cores by using nickelocene as the reactants through the inorganic-organic competitive coating strategy.

Supplementary Figure 18. The CV curves of the hollow gradient-structured Fe_3O_4@C (HG-Fe_3O_4@C) electrode.

Supplementary Figure 19. The lithium-storage characteristic of gradient-structured Fe_3O_4@C nanospheres at low current density of 0.1 C.

Supplementary Figure 20. Two kinds of commonly used nanostructures for anode.

Supplementary Figure 21. Morphology characterization of the hollow island-type C@Fe_3O_4 (HI-C@Fe_3O_4) nanospheres.

Supplementary Figure 22. *Ex-situ* TEM images of the hollow gradient-structured Fe_3O_4@C nanospheres at different states of charge and discharge during the 5th cycle at current density of 0.2 A g^{-1}.

Supplementary Figure 23. *Ex-situ* TEM images of the hollow gradient-structured Fe_3O_4@C (HG-Fe_3O_4@C) nanospheres at a current density of 0.2 A g^{-1}.

Supplementary Figure 24. *Ex-situ* TEM images of the gradient-structure of Fe_3O_4@C nanospheres after 10000 cycles at 10 A g^{-1}.

Supplementary Figure 25. Cycling performances of the yolk-shell Fe_3O_4@C (YS-Fe_3O_4@C) electrode under a high current density of 10 A g^{-1}.

Supplementary Figure 26. Cycling performances of the hollow hybrid Fe_3O_4/C (HH-Fe_3O_4/C) electrode under a high current density of 10 A g^{-1}.

Supplementary Figure 27. *Ex-situ* SEM images of the hollow gradient-structured Fe_3O_4@C electrode and typical nanospheres.

Supplementary Figure 28. Morphology of the yolk-shell Fe_3O_4@C (YS-Fe_3O_4@C) electrode after 1000 cycles under a current density of 10 A g^{-1}.

Supplementary Figure 29. Morphology of hollow hybrid Fe_3O_4/C (HH-Fe_3O_4/C) electrode after 1000 cycles under a current density of 10 A g^{-1}.

Supplementary Figure 30. Asymmetric charging and discharging performance hollow gradient-structured Fe_3O_4@C (HG-Fe_3O_4@C) electrodes**.**

Supplementary Figure 31. The performance of the gradient-structured Fe_3O_4@C nanospheres anodes with high mass loading of 5, 10, and 20 mg/cm^2 at 10 A g^{-1}.

Supplementary Table 1. Comparison of the gradient-structured Fe_3O_4@C nanospheres with previous works.

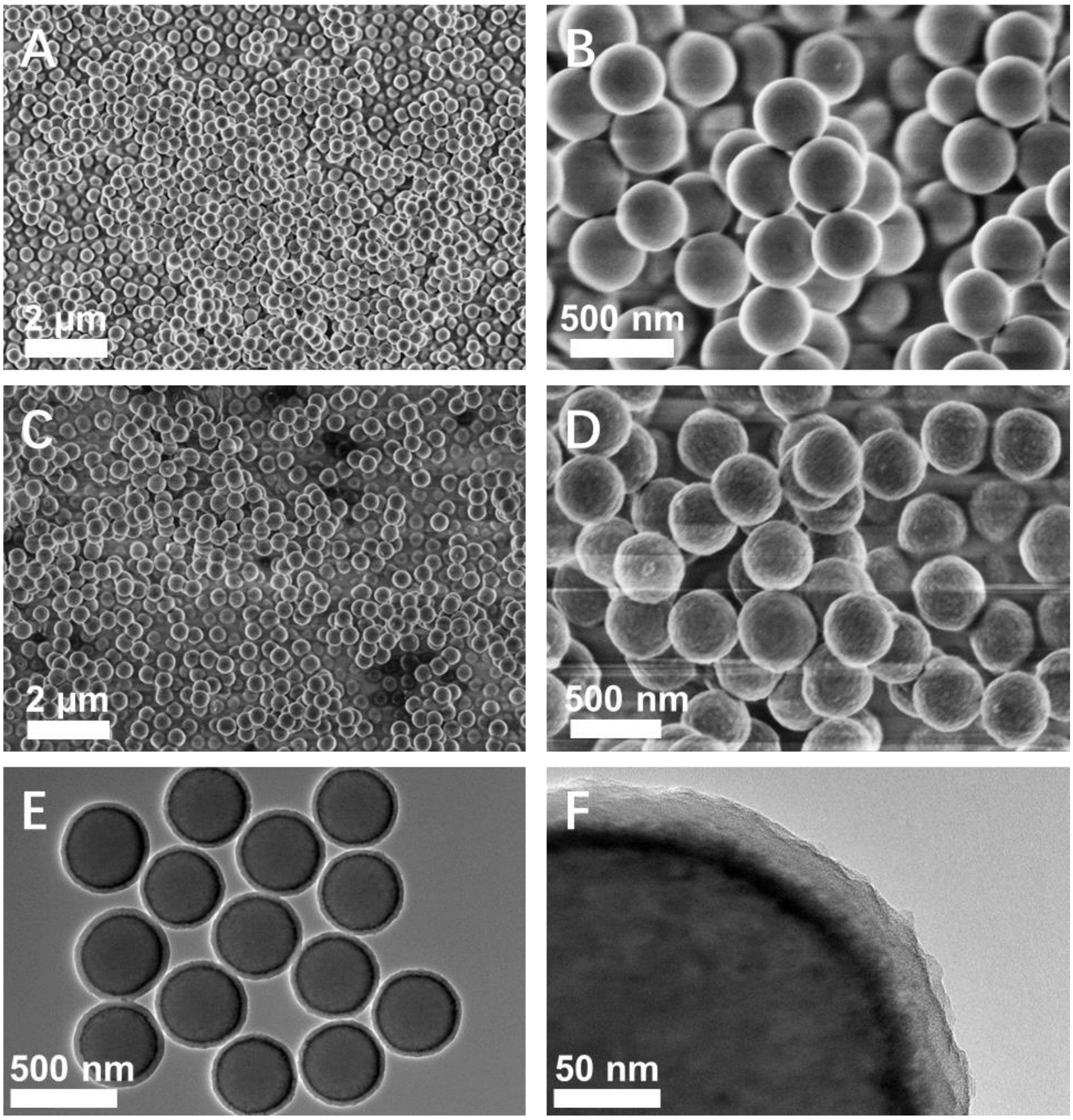

Supplementary Figure 1. Morphology characterization of the pure colloidal SiO_2 cores and as-made gradient-structured nanospheres (SiO_2@G-Fe_3O_4@C) after the inorganic-organic competitive coating strategy at 210 °C under the solvothermal condition. (A, B) SEM images of the colloidal SiO_2 nanospheres with a particle size of 350 nm; (C, D) SEM images of the as-made gradient-structured SiO_2@G-Fe_3O_4@C nanospheres with a particle size of 420 nm.

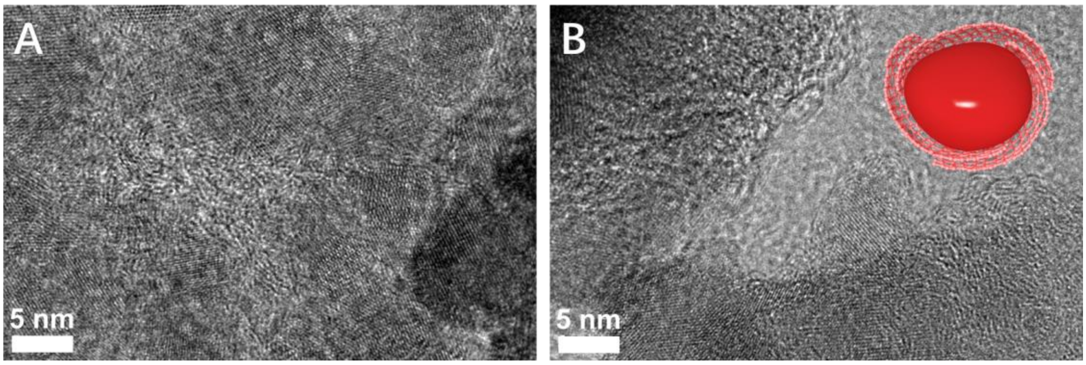

Supplementary Figure 2. HRTEM images of a broken hollow gradient-structured Fe_3O_4@C nanosphere after mechanical milling for 1 h. (A) The central and (B) edge regions of the broken hollow gradient-structured Fe_3O_4@C nanospheres. It can be seen that every Fe_3O_4 nanocrystals are conformably encapsulated in ultrathin graphitic carbon layers. The inset of (B) is the model diagram of encapsulated Fe_3O_4 nanocrystal.

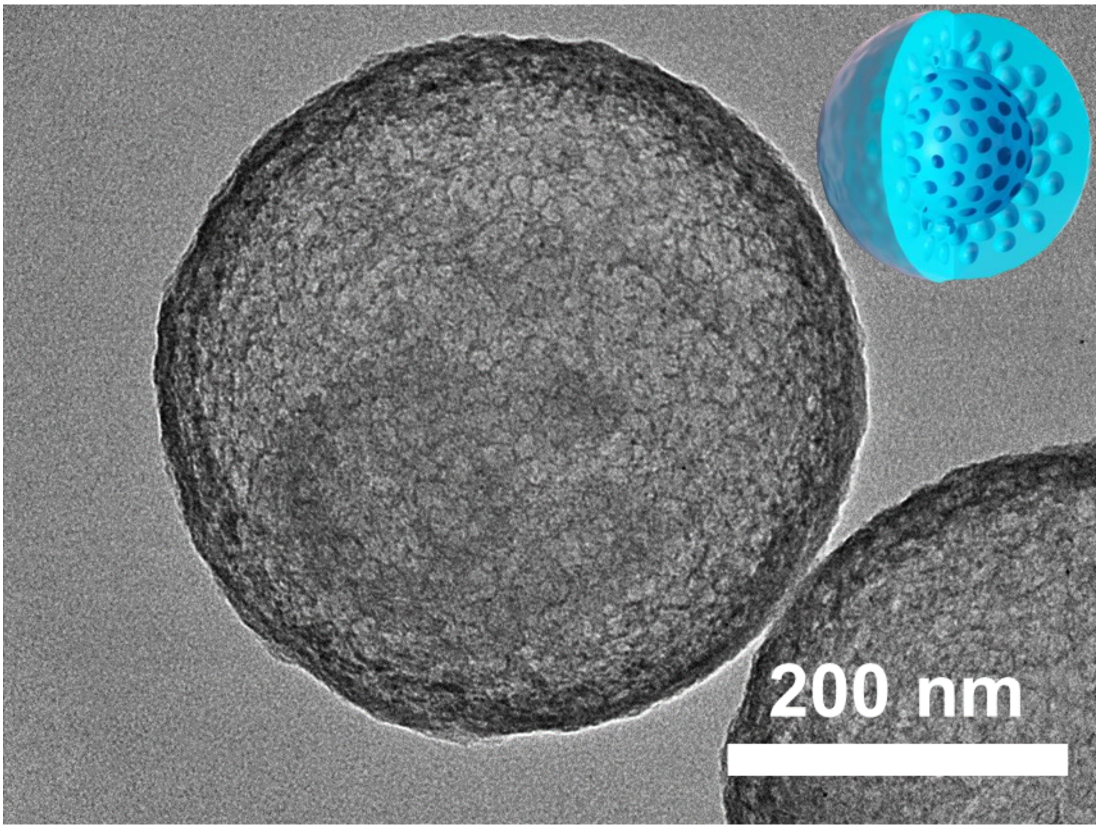

Supplementary Figure 3. The TEM image of the hollow carbon nanosphere obtained by etching the hollow gradient-structured Fe_3O_4@C nanopheres with 0.1 M HF solution to remove of all Fe_3O_4 nanoparticles. It can be seen that the inner surface of the obtained hollow carbon nanospheres is pitted with craters, obviously indicating that the Fe_3O_4 nanoparticles are gradient embedded in the inner walls of the protective layer matrixes. The inset is the model diagram of hollow carbon nanosphere.

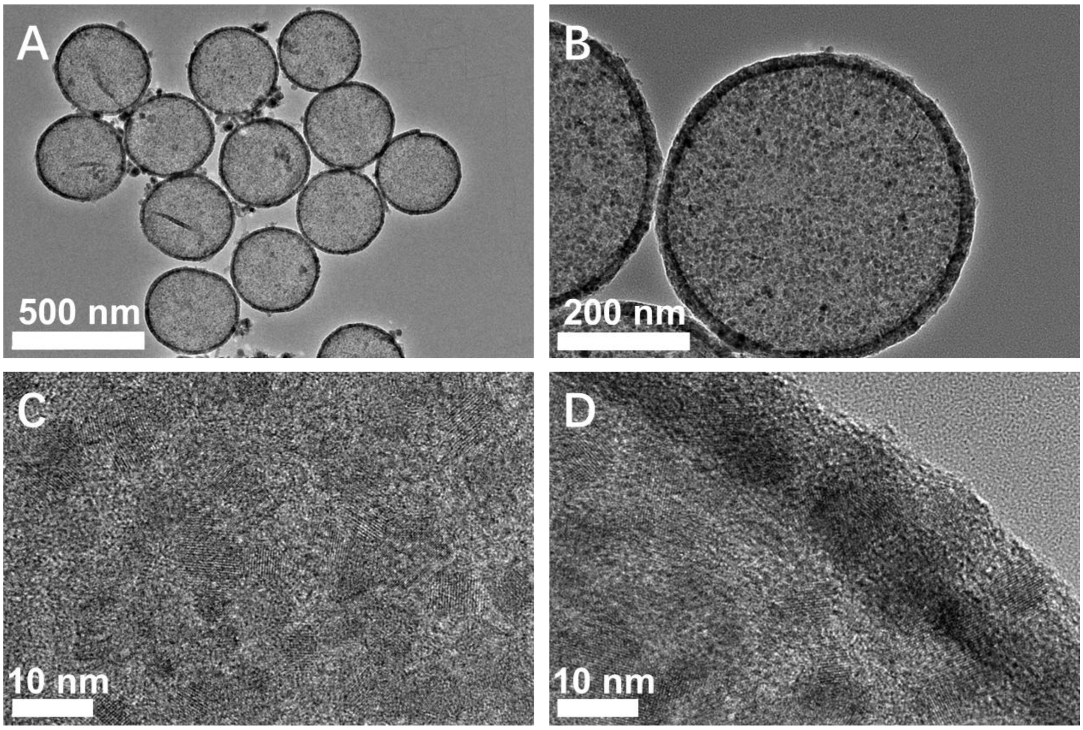

Supplementary Figure 4. TEM images of the hollow hybrid Fe_3O_4/C (HH-Fe_3O_4/C) nanospheres obtained by etching the as-made SiO_2@G-Fe_3O_4@C nanospheres with 1 M NaOH before annealing to remove the outer amorphous carbonaceous layers and colloidal SiO_2 nanosphere cores. (A) Several nanospheres; (B) A typical nanosphere; (C) the central and (D) edge region of the typical nanosphere. It can be seen that the Fe_3O_4 nanoparticles are gradient assembled to form the hollow-hybrid Fe_3O_4/C nanospheres (HH-Fe_3O_4/C), further revealing the existence of gradient distribution of Fe_3O_4 nanoparticles in the products.

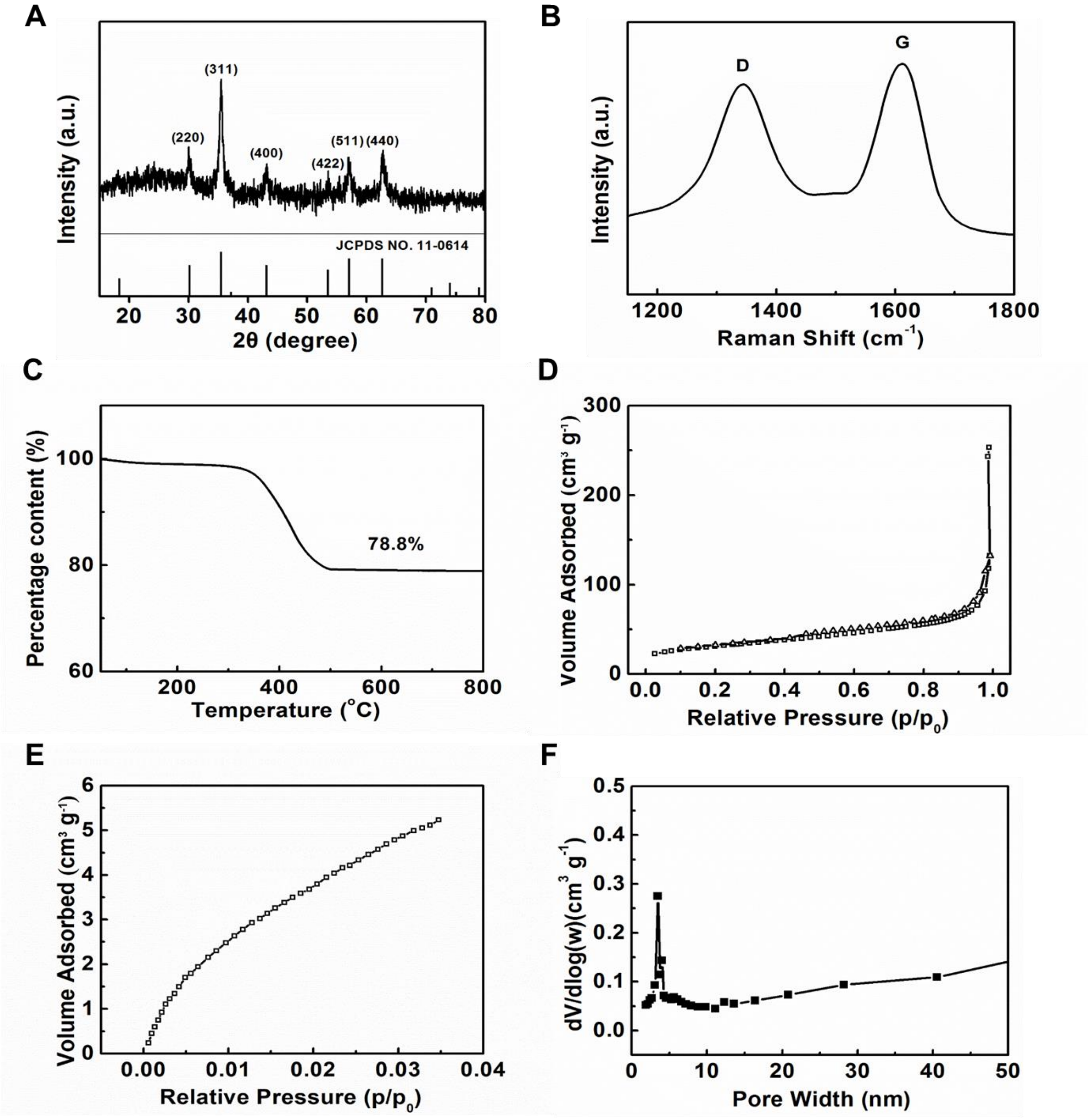

Supplementary Figure 5. Superficial characteristics of the hollow gradient-structured Fe_3O_4@C nanospheres. (A) XRD pattern; (B) Raman spectra; (C) TGA curve; (D) nitrogen adsorption-desorption isotherms; (E) Adsorption curve of carbon dioxide; and (F) the pore size distribution.

According to the theory of X-ray diffraction, when the particle size is less than 100 nm, the width of the diffraction peak becomes obvious with the decrease of particle size. Considering the absorption effect of the sample and the influence of structure on the diffraction pattern, the average crystallite sizes of Fe_3O_4 can be calculated by the Debye-Scherrer formula:

$$D = K\lambda / \beta \cos \theta$$

Where D is the crystallite diameter perpendicular to the crystal plane, K is the Scherrer constant (usually 0.89), λ is the incident X-ray wavelength ($\lambda = 0.15406$ nm), and θ is Bragg diffraction angle (°), β is half peak width (rad) of diffraction peak. Input the data from the diffraction peak of (311) crystal plane for calculation ($\beta = 1.55° \approx 0.027$ rad, $\theta = 17.726°$), therefore D is equal to ~5.33 nm. This size is matched with TEM data.

The Raman spectra of gradient-structured Fe_3O_4@C nanospheres exhibit two peaks at approximately 1345 and 1613 cm^{-1}, which are characteristic of the breathing mode of aromatic rings (the D band) and the bond stretching of the sp_2 carbon (the G band), respectively. The G band of the sample shift to a higher wave number of 1613 cm^{-1} compared with the standard wave number of graphite single crystal (1575 cm^{-1}), indicating that a large amount of disordered graphite-like carbon is presented in the gradient-structured Fe_3O_4@C nanospheres.

Let the mass fraction of Fe_3O_4 in the Fe_3O_4@C to be x. If the carbon is completely combusted as the temperatures approach 700 °C, it would get this equation: 3*x/231.54=(1-21.2%)*2/159.69. Therefore x = 76.2%. The carbon content in the gradient-structured Fe_3O_4@C nanospheres could be estimated to be about 23.8% from the TGA curves. The BET surface area was calculated to be ~ 141 m^2 g^{-1} with a pore diameter of ~ 4.0 nm.

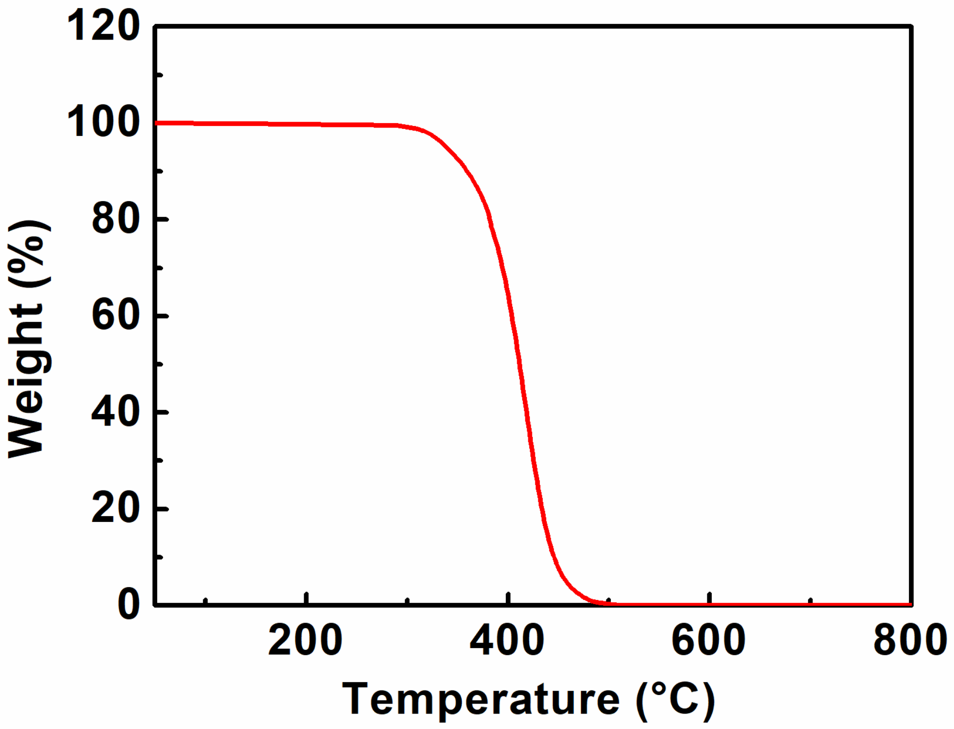

Supplementary Figure 6. The TGA curve of the sample after etching by HF for the removal of Fe_3O_4. The result shows no presence of remaining impurities in the gradient-structured Fe_3O_4@C nanospheres.

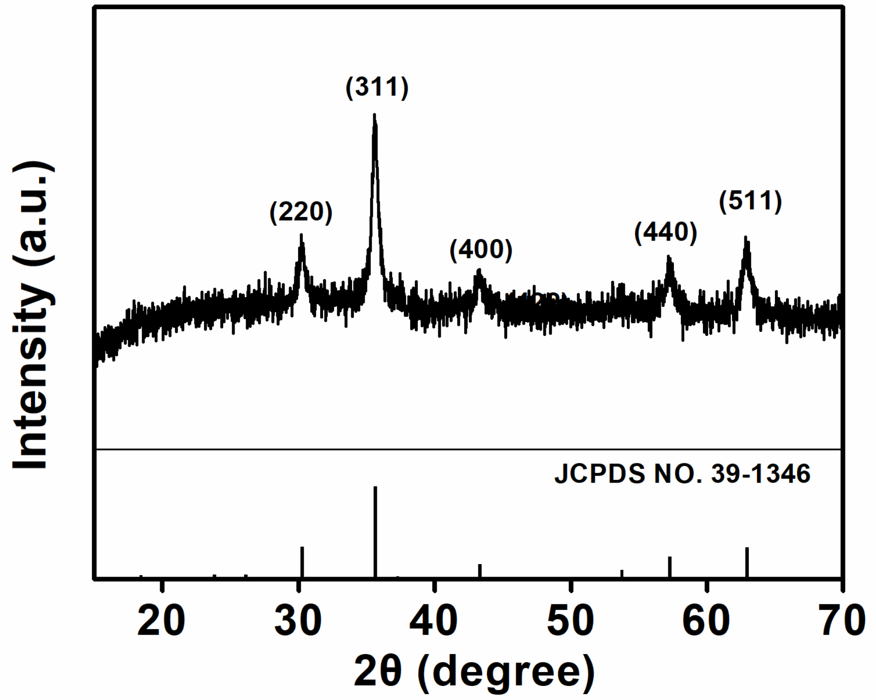

Supplementary Figure 7. The XRD pattern of the red product after TG analysis process. XRD result shows that all diffraction peaks can be well assigned to pure Fe_2O_3 (JCPDS card no.39-1346).

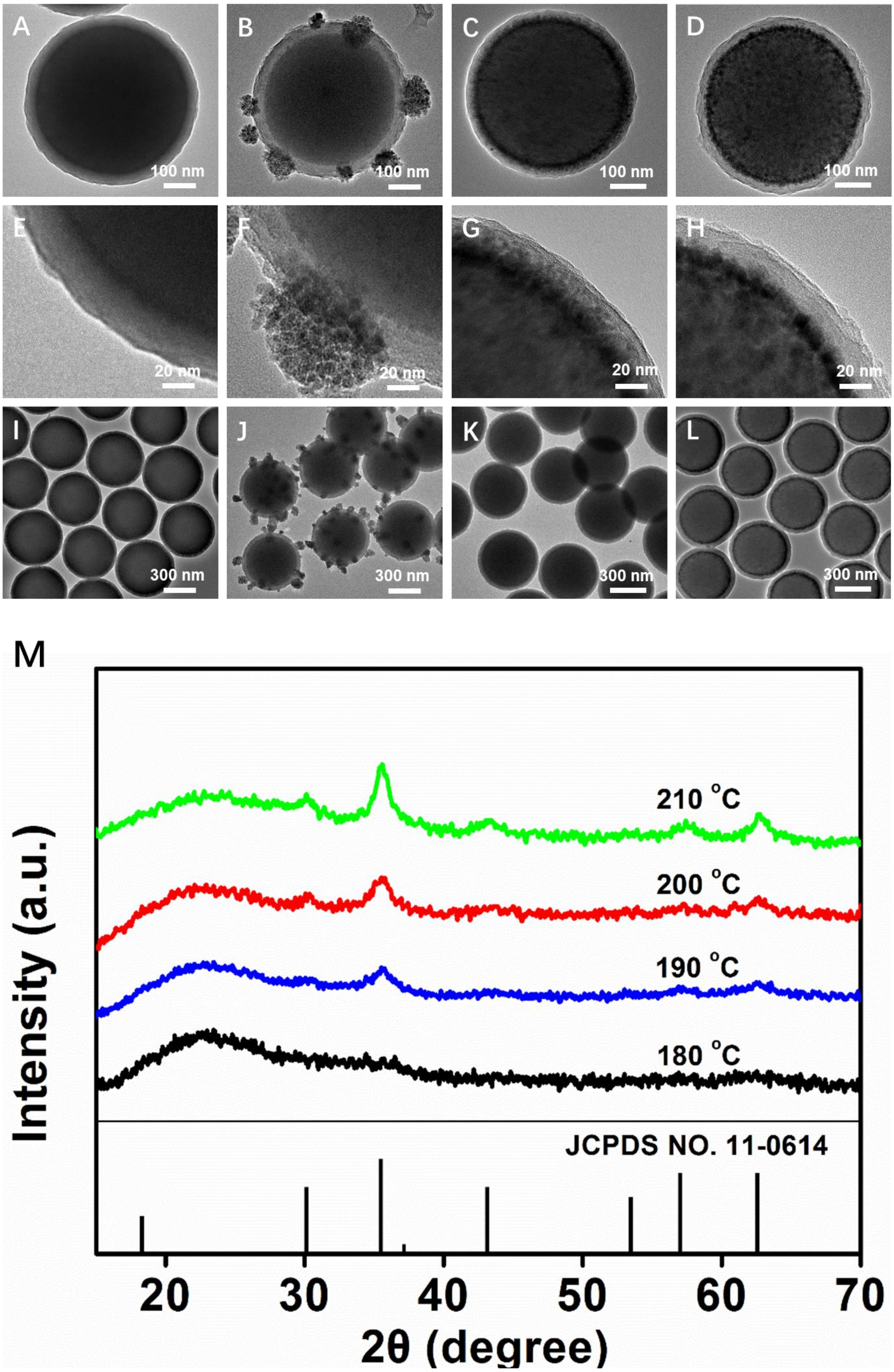

Supplementary Figure 8. TEM images and XRD patterns of the products prepared by different reaction temperature through the inorganic-organic competitive

coating strategy. (A, E, I) TEM images of the products obtained at 180 °C; (B, F, G) TEM images of the products obtained at 190 °C; (C, J, K) TEM images of the products obtained at 200 °C; (D, H, L) TEM images of the products obtained at 210 °C; and (M) XRD patterns of the products obtained by solvothermal reaction at 210 °C, 200 °C, 190 °C, and 180 °C. These results clearly reveal that the morphology of the coating shells is much relied on the reaction temperatures.

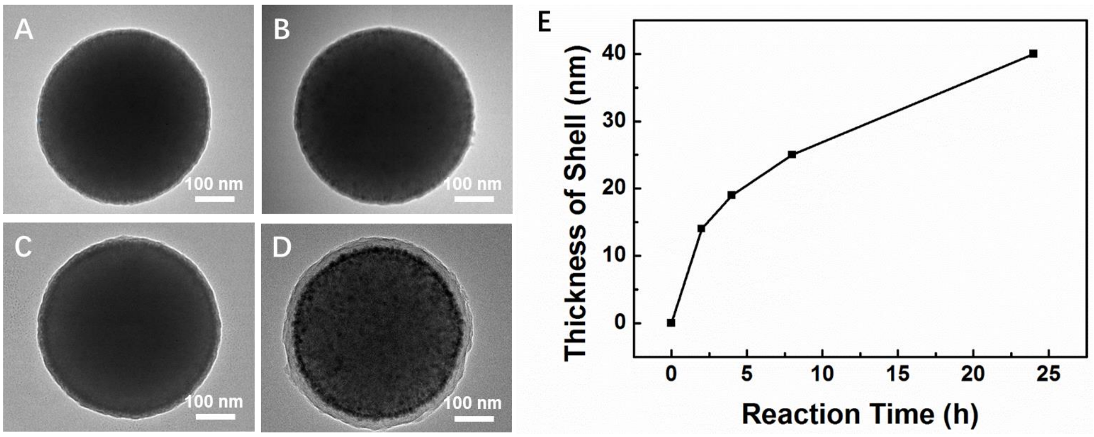

Supplementary Figure 9. TEM images and thickness curve of time-dependent experiment under the solvothermal condition of 210 ℃. (A) TEM images of the product after 2 h; (B) TEM images of the product after 4 h; (C) TEM images of the product after 8 h; (D) TEM images of the product after 24 h; and (E) thickness of the gradient-structured Fe_3O_4@C shell during the solvothermal reaction. At the beginning of reaction, the Fe_3O_4 nanoparticles covered by ultrathin carbon layer are first deposited on the SiO_2 cores to form a Fe_3O_4-rich layer. With increasing reaction time, the thickness of the Fe_3O_4-rich layers is increased, and then encapsulated by a pure polymer carbon layer. It can be clearly observed that there is a process of explosive nucleation at the beginning of the solvothermal reaction.

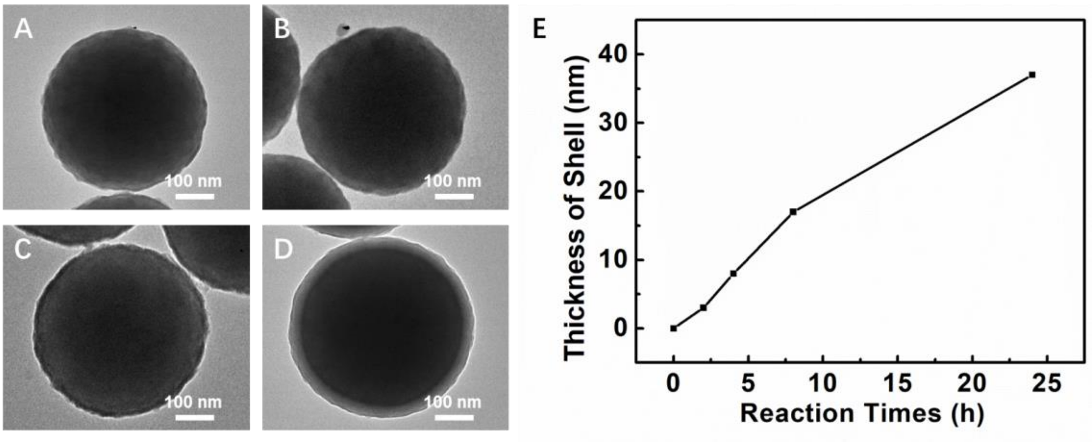

Supplementary Figure 10. TEM images and thickness curve of time-dependent experiment under the solvothermal condition of 180 °C. (A) TEM images of the product after 2 h; (B) TEM images of the product after 4 h; (C) TEM images of the product after 8 h; (D) TEM images of the product after 24 h; and (E) the thickness of the pure amorphous carbonaceous layer during the solvothermal reaction of 180 °C. It can be clearly observed that the thickness of the polymer carbon layers is mainly dependent on reaction times.

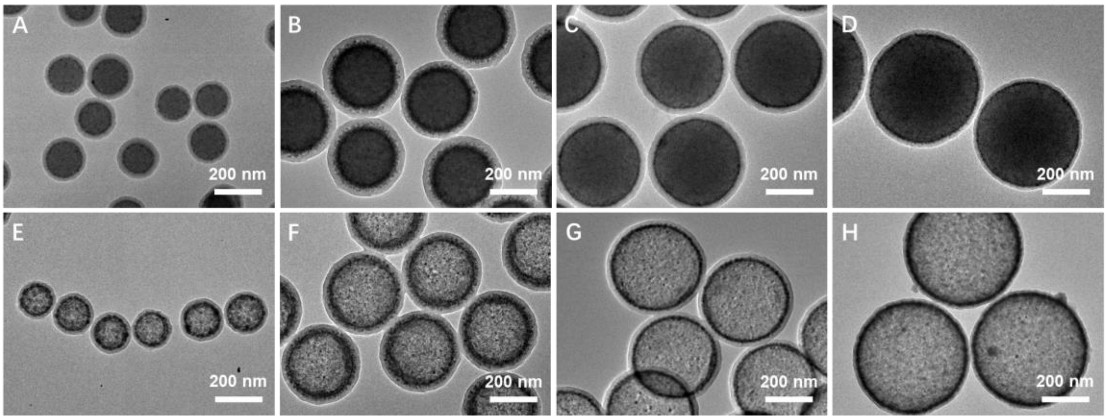

Supplementary Figure 11. TEM images of the as-made gradient-structured nanospheres (SiO_2@G-Fe_3O_4@C) with a controllable particle size from 150 to 500 nm and their derived hollow gradient-structured Fe_3O_4@C nanospheres. (A-D) the core-shell SiO_2@G-Fe_3O_4@C nanospheres with average SiO_2 core size: (A) 120 nm; (B) 210 nm; (C) 330 nm; and (D) 450 nm. (E-H) their derived hollow gradient-structured Fe_3O_4@C (HG-Fe_3O_4@C) nanospheres, respectively. It can be seen that the void sized of the hollow gradient nanospheres can be tuned from ~120 to 450 nm by using colloidal SiO_2 cores with different diameters.

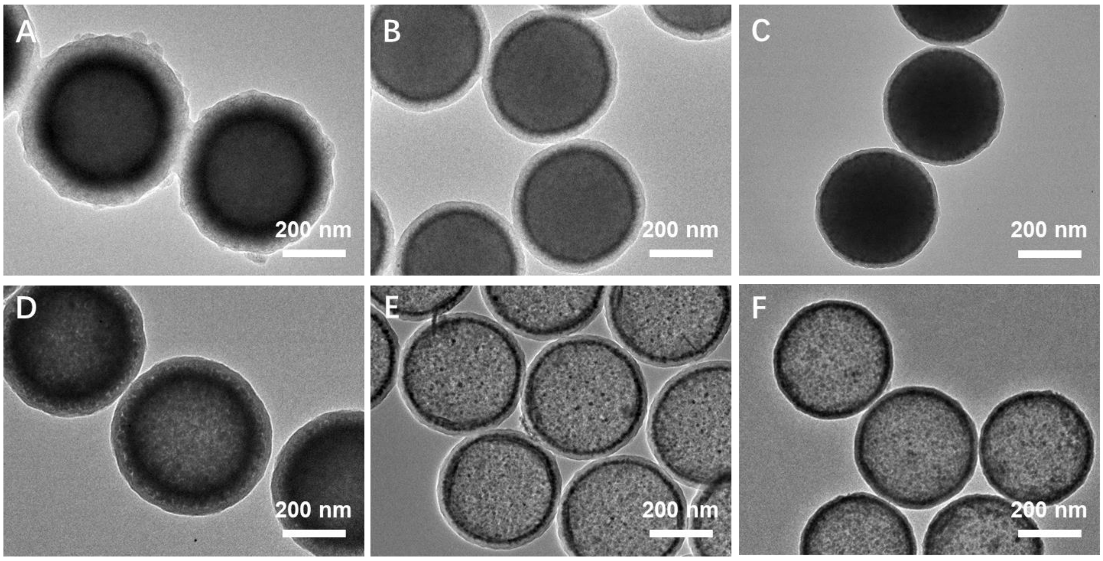

Supplementary Figure 12. TEM images of the as-made gradient-structured nanospheres (SiO_2@G-Fe_3O_4@C) with varied shell thicknesses from 20 to 80 nm and their derived hollow gradient-structured Fe_3O_4@C nanospheres. The thickness of (A, D) 80 nm; (B, E) 40 nm; and (C, F) 20 nm. It can be seen that the thicknesses of the gradient-structured Fe_3O_4@C shells can be tuned from ~ 20 to 80 nm by controlling the amount of the colloidal SiO_2 suspension from low (0.0375 g of SiO_2 nanospheres) to high (0.3 g of SiO_2 nanospheres) during the solvothermal reaction at 210 °C.

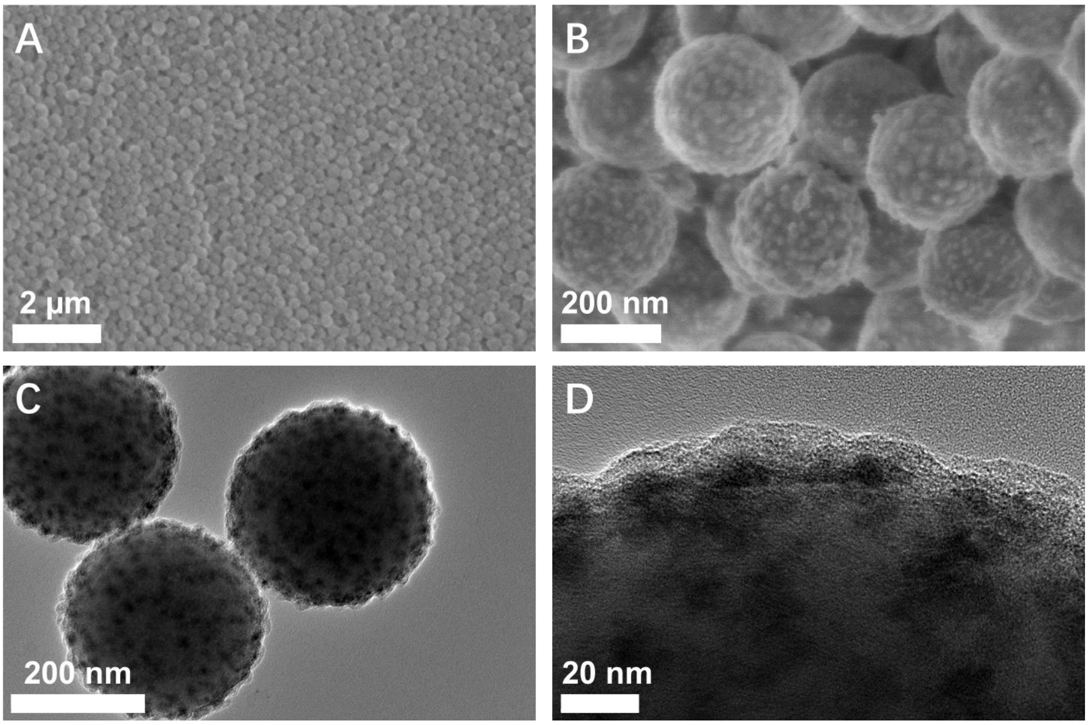

Supplementary Figure 13. Morphology characterization of the strawberry-like Fe_3O_4@C (S-Fe_3O_4@C) shells coating on the excessive colloidal SiO_2 cores. (A, B) SEM images and (C, D) TEM images of the strawberry-like SiO_2@S-Fe_3O_4@C nanospheres. In the process of tuning the thickness of Fe_3O_4@C shells, when the amount of the colloidal SiO_2 suspension was high (4 ml, 0.6 g), the generated Fe_3O_4 nanoparticles were not enough to cover the entire surface of the colloidal SiO_2, thus resulting in the formation of a strawberry-like shell.

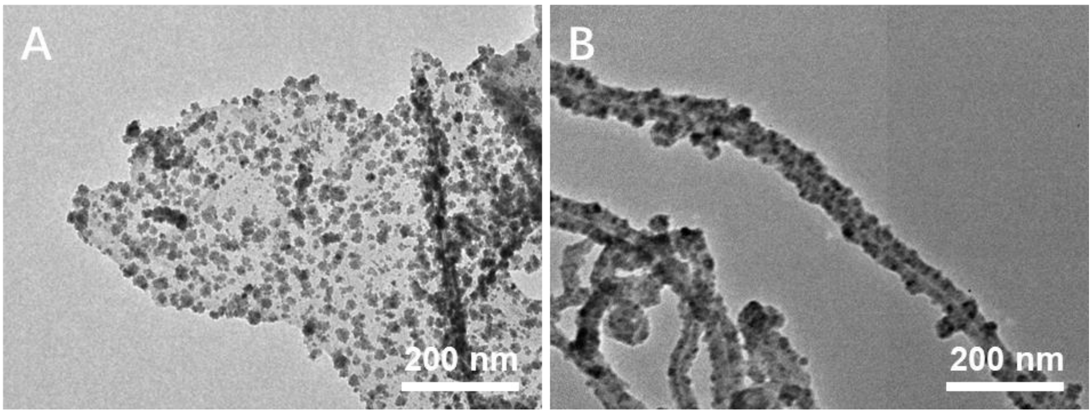

Supplementary Figure 14. TEM images of the strawberry-like Fe_3O_4@C (S-Fe_3O_4@C) shells coating on carbon substrate cores with excessive interface. (A) Graphite oxide coated by strawberry-like Fe_3O_4@C shells (GO@S-Fe_3O_4@C); and (B) carbon nanotubes coated by strawberry-like Fe_3O_4@C shells (CNTs@S-Fe_3O_4@C). Graphite oxide and CNTs possess a large specific surface area. In the process of coating the gradient-structured Fe_3O_4@C shells, if the SiO_2 core was replaced by graphene or CNTs, the generated Fe_3O_4 nanoparticles could not be enough to cover the entire surface of the graphene or CNTs, thus resulting in the formation of a strawberry-like shell.

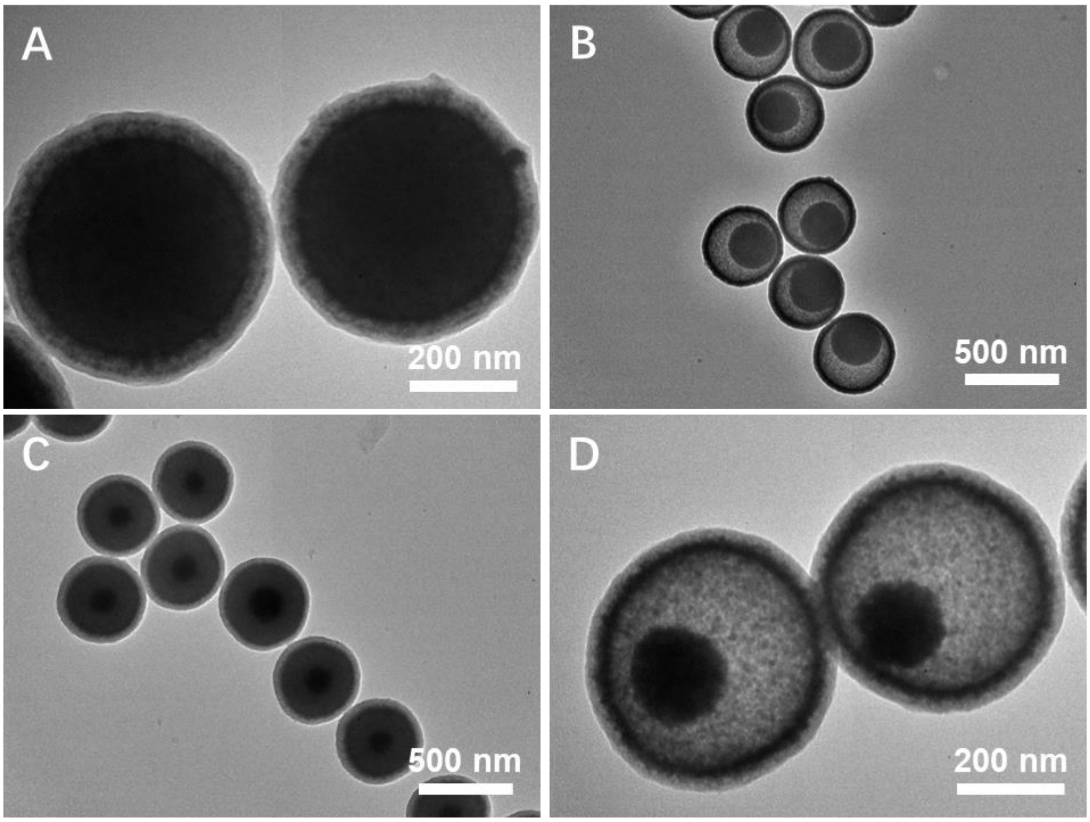

Supplementary Figure 15. TEM images of the functional silica nanosphere cores coated by the gradient-structured Fe_3O_4@C shells and their derived yolk-shell structured nanospheres. (A) the colloidal SiO_2 core coated by the gradient-structured Fe_3O_4@C shell for two times; (B) yolk-shell SiO_2@void@G-Fe_3O_4@C obtained by etching the SiO_2@G-Fe_3O_4@C core-shell nanospheres with 1.0 M NaOH aqueous solution for 6 h to partially remove the colloidal SiO_2 cores; (C) the core-shell structured Fe_3O_4@SiO_2 nanospheres coated by the gradient-structured Fe_3O_4@C shells (Fe_3O_4@SiO_2@G-Fe_3O_4@C); and (D) their derived yolk-shell structured Fe_3O_4@void@G-Fe_3O_4@C nanospheres. In the inorganic-organic competitive coating strategy, the colloidal SiO_2 cores can be replaced by the SiO_2@G-Fe_3O_4@C or Fe_3O_4@SiO_2 nanospheres, implying that this coating strategy is versatile and can be easily extended to prepare other new core-shell and hollow gradient structures.

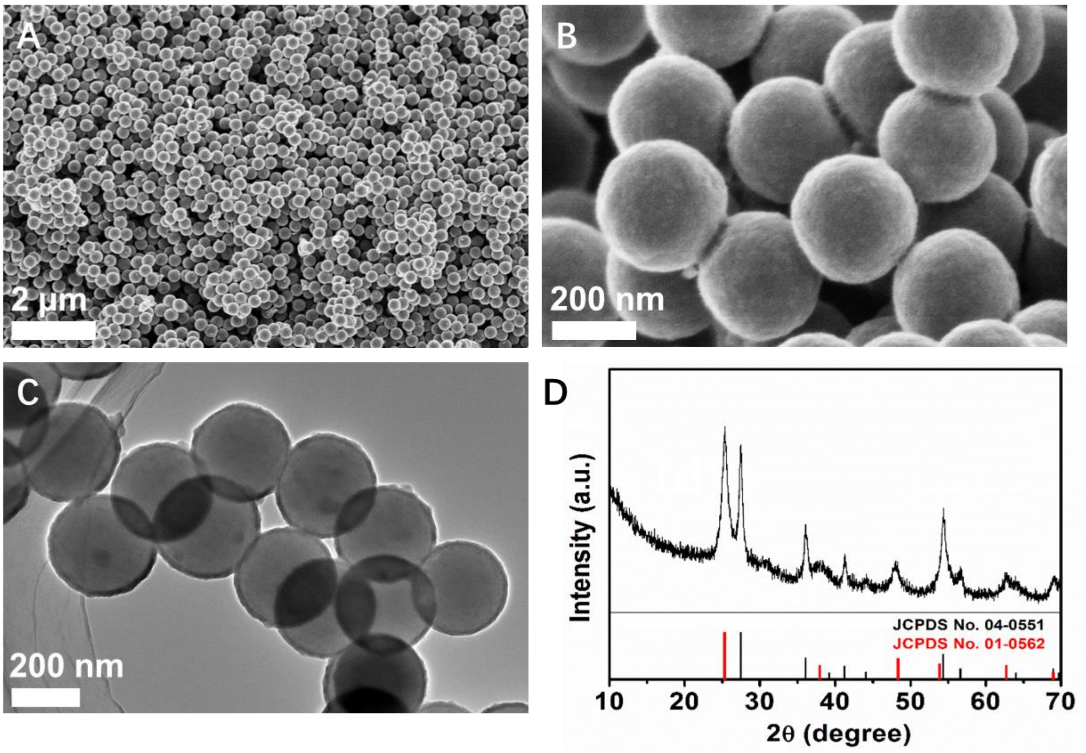

Supplementary Figure 16. Characterization of the TiO_2@C (G-TiO_2@C) shells coating on the colloidal SiO_2 cores by using titanocene as the reactants through the inorganic-organic competitive coating strategy. (A, B) SEM image of the uniform SiO_2@G-TiO_2@C nanospheres; (C) TEM image of the core-shell SiO_2@G-TiO_2@C nanospheres, and (D) XRD pattern of the SiO_2@G-TiO_2@C nanospheres.

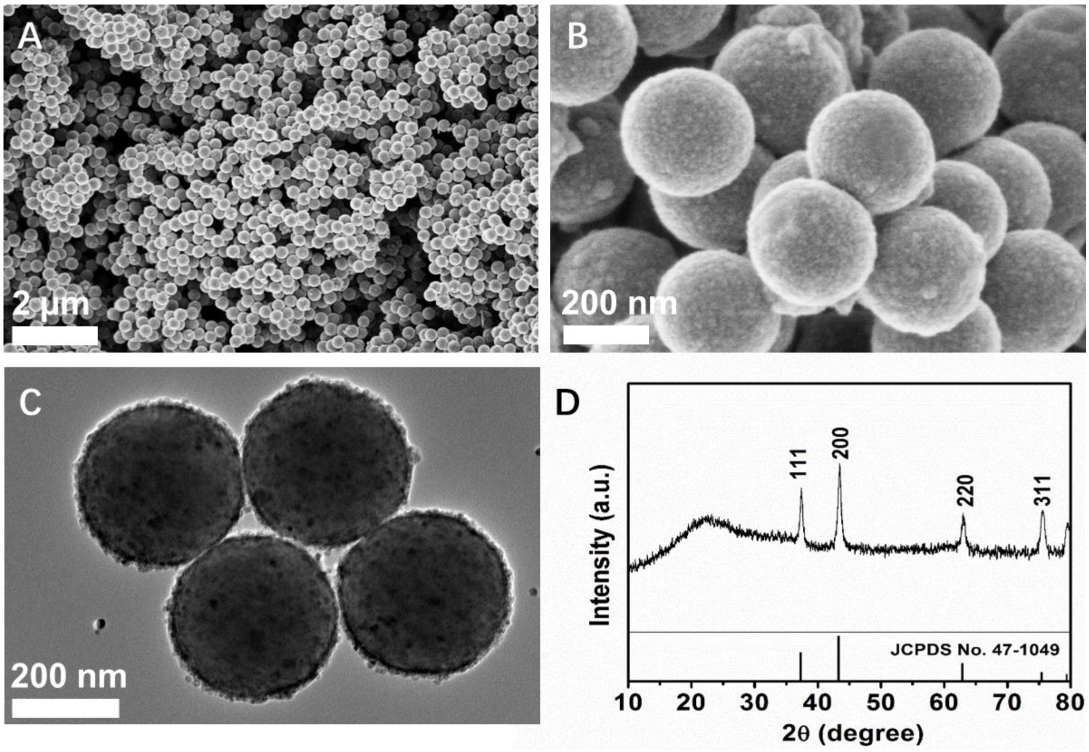

Supplementary Figure 17. Characterization of the NiO@C (G-NiO@C) shells coating on the colloidal SiO_2 cores by using nickelocene as the reactants through the inorganic-organic competitive coating strategy. (A, B) SEM image of the uniform SiO_2@G-NiO@C nanospheres; (C) TEM image of the core-shell SiO_2@G-NiO@C nanospheres, and (D) XRD pattern of the SiO_2@G-NiO@C nanospheres.

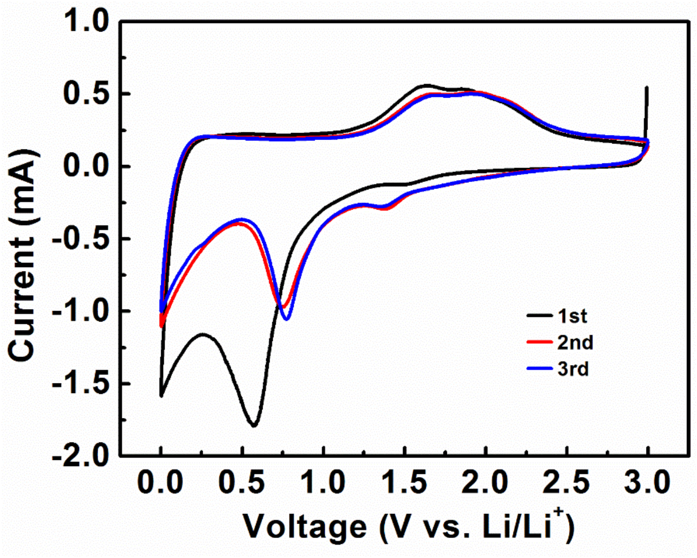

Supplementary Figure 18. The CV curves of the hollow gradient-structured Fe_3O_4@C (HG-Fe_3O_4@C) electrode. The electrode was prepared by mixed active materials (HG-Fe_3O_4@C), binder (poly(vinylidene fluoride)) and conductive agent (acetylene black) with a weight ratio of 8:1:1.

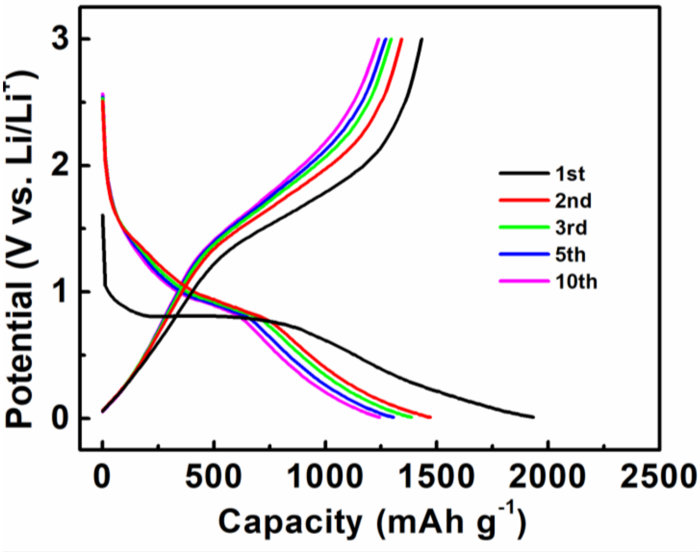

Supplementary Figure 19. The lithium-storage characteristic of gradient-structured Fe_3O_4@C nanospheres at low current density of 0.1 C. Based on Fe_3O_4, 1 C = 926 mA g^{-1}.

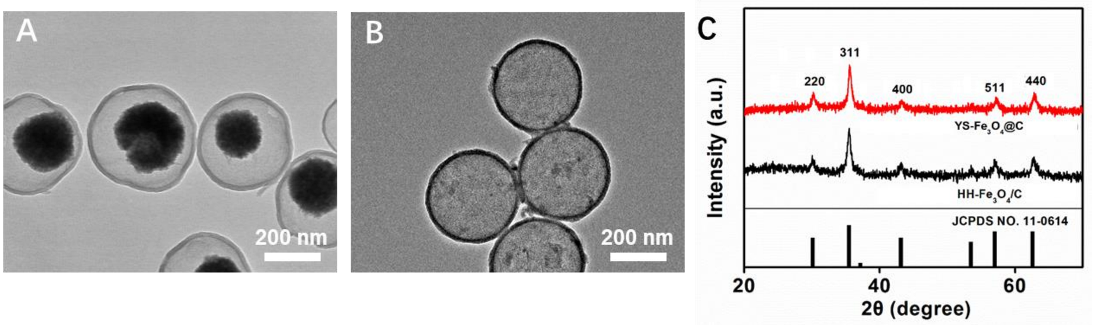

Supplementary Figure 20. Two kinds of commonly used nanostructures for anode. (A) TEM images of the yolk-shell structured Fe_3O_4@C (YS-Fe_3O_4@C) nanospheres; (B) TEM images of the hollow hybrid Fe_3O_4/C (HH-Fe_3O_4@C); and (C) XRD patterns of the yolk-shell structured Fe_3O_4@C and hybrid hollow Fe_3O_4@C nanospheres. X-ray diffraction (XRD) peaks of the hollow hybrid Fe_3O_4/C and yolk-shell structured Fe_3O_4@C nanospheres can be well assigned magnetite with *Fd3m* symmetry (JCPDS: NO.11-0614).

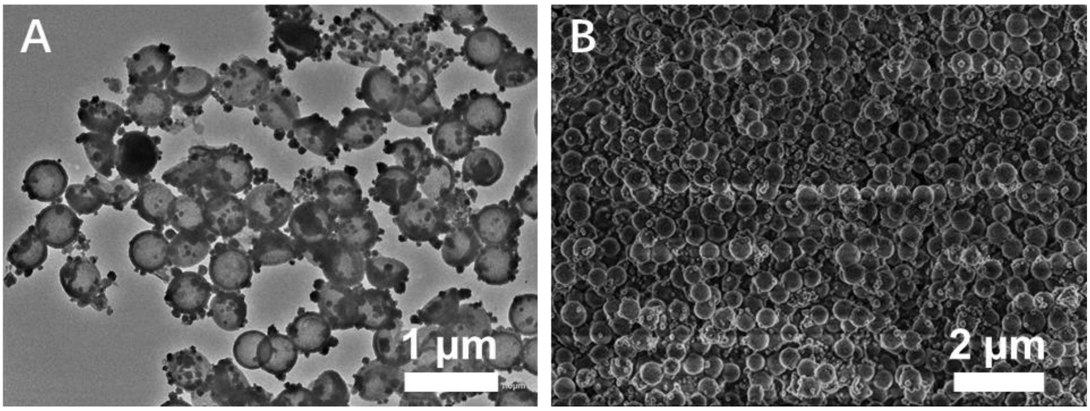

Supplementary Figure 21. Morphology characterization of the hollow island-type $C@Fe_3O_4$ (HI-$C@Fe_3O_4$) nanospheres. (A) TEM and (B) SEM images of the hollow island-type $C@Fe_3O_4$ nanospheres. It was derived from the products of 190 °C through inorganic-organic competitive coating strategy.

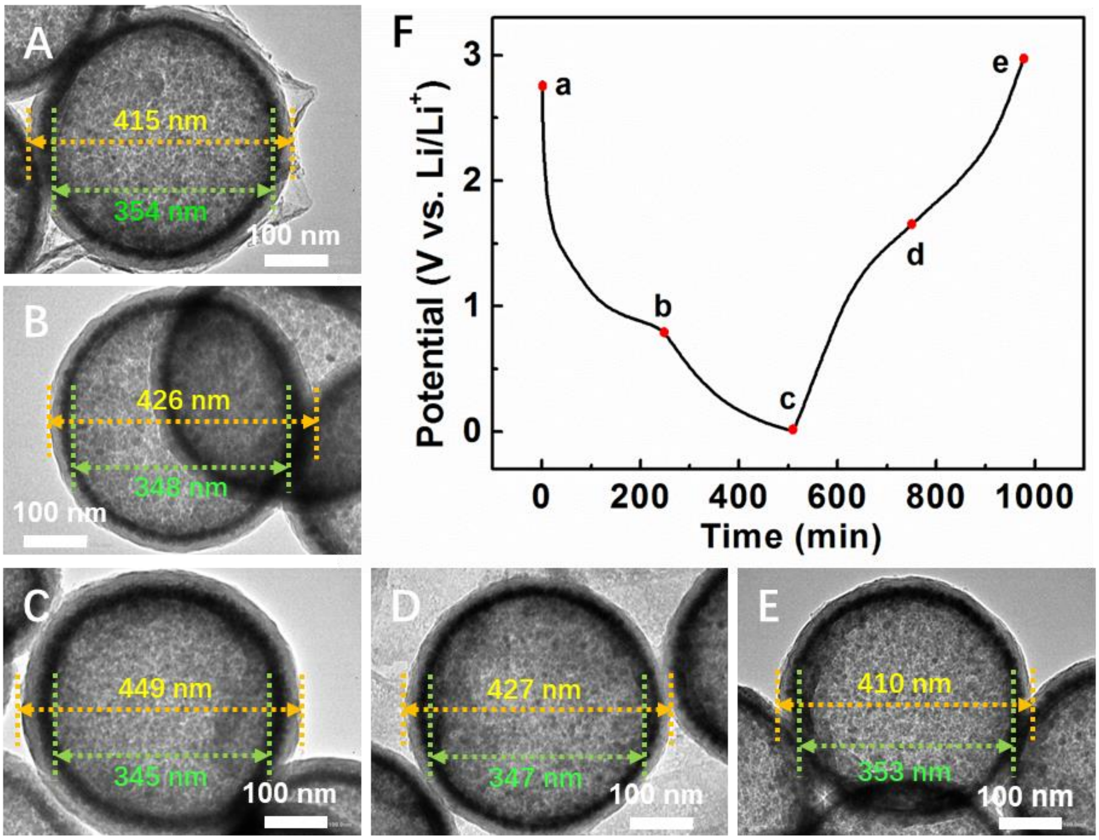

Supplementary Figure 22. ***Ex-situ*** **TEM images of the hollow gradient-structured Fe_3O_4@C nanospheres at different states of charge and discharge during the 5th cycle at current density of 0.2 A g^{-1}.** (A) Without discharge; (B) half discharge; (C) full discharge, (D) half charge; (E) full charge; and (F) corresponding discharge and charge curves.

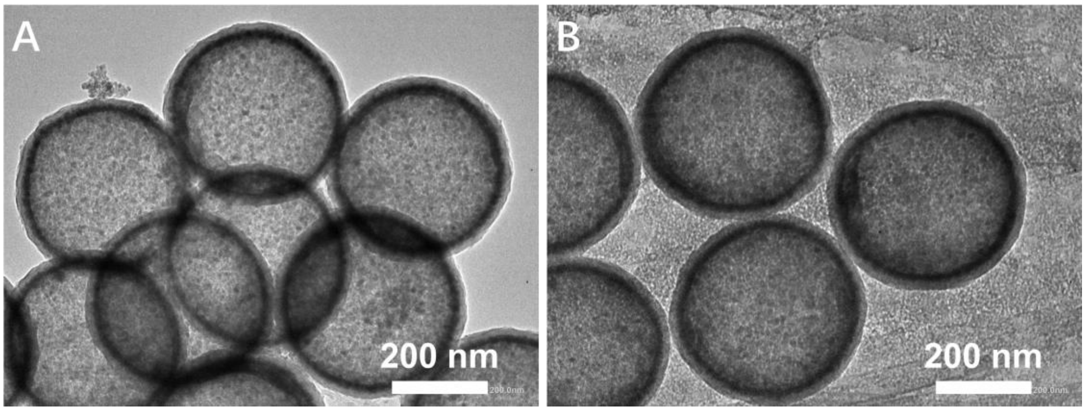

Supplementary Figure 23. ***Ex-situ*** **TEM images of the hollow gradient-structured Fe_3O_4@C (HG-Fe_3O_4@C) nanospheres at a current density of 0.2 A g^{-1}.** (A) After 30 cycles; and (B) after 100 cycles.

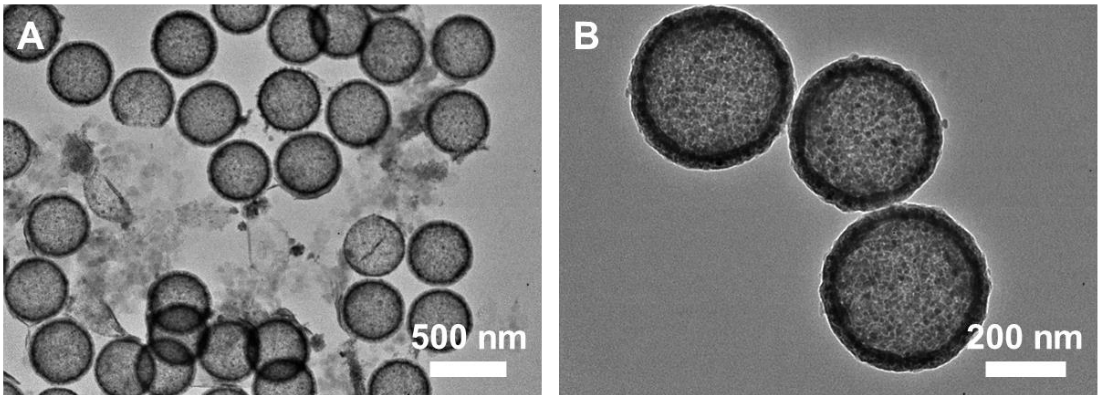

Supplementary Figure 24. ***Ex-situ*** **TEM images of the gradient-structure of Fe_3O_4@C nanospheres after 10000 cycles at 10 A g^{-1}.** (A) Several nanospheres; and (B) Typical nanospheres.

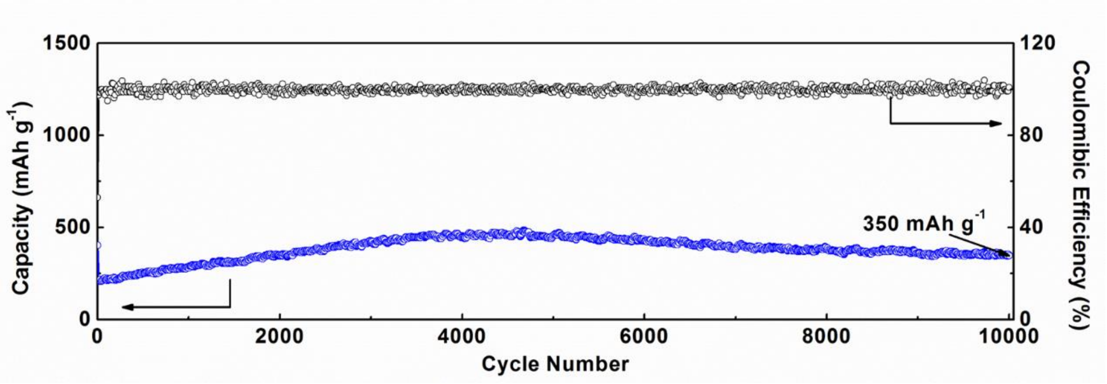

Supplementary Figure 25. Cycling performances of the yolk-shell structured Fe_3O_4@C (YS-Fe_3O_4@C) electrode under a high current density of 10 A g^{-1}. After 10000 cycles, it can deliver a capacity of 350 mAh g^{-1} with a low Coulombic efficiency.

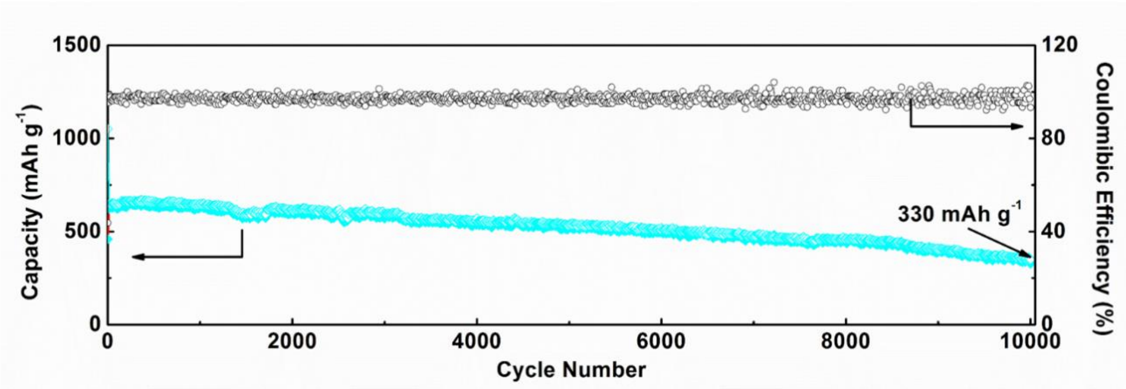

Supplementary Figure 26. Cycling performances of the hollow hybrid Fe_3O_4/C (HH-Fe_3O_4/C) electrode under a high current density of 10 A g^{-1}. After 10000 cycles, it can deliver a capacity of 330 mAh g^{-1} with a low Coulombic efficiency.

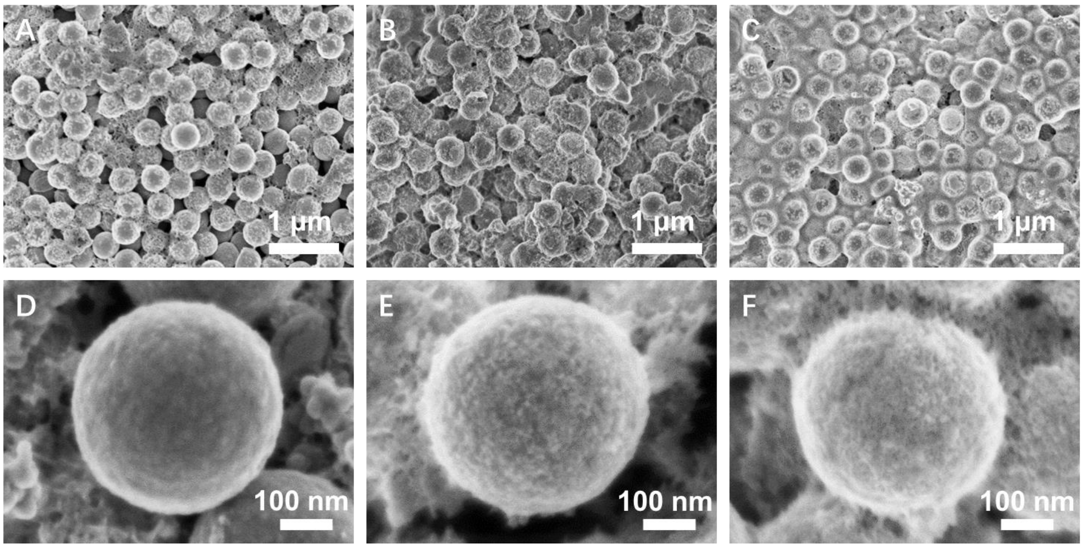

Supplementary Figure 27. ***Ex-situ*** **SEM images of the hollow gradient-structured Fe_3O_4@C electrode and typical nanospheres.** (A, D) the fresh one; (B, E) after 100 cycles at 10 Ag^{-1}; (C, F) after 1000 cycles at 10 Ag^{-1}.

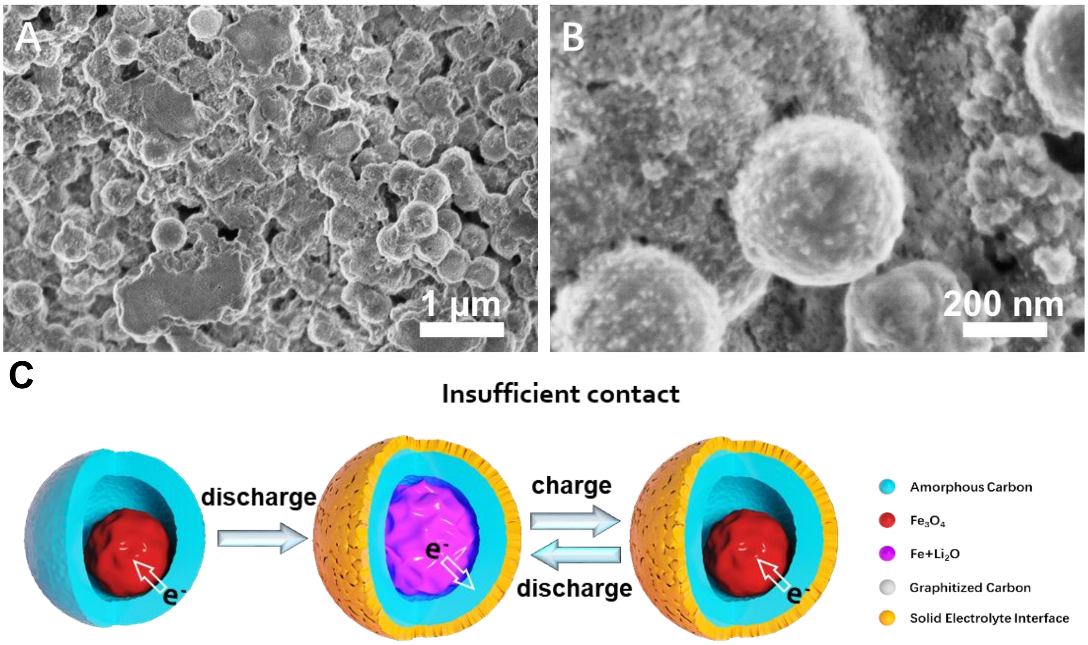

Supplementary Figure 28. Morphology of the yolk-shell structured Fe_3O_4@C (YS-Fe_3O_4@C) electrode after 1000 cycles under a current density of 10 A g^{-1}. (A) SEM image of the yolk-shell structured YS-Fe_3O_4@C electrode surface; (B) SEM image of the typical YS-Fe_3O_4@C nanospheres on the electrode; (C) Schematic illustrations for the structural change of the yolk-shell structured Fe_3O_4@C nanospheres during fast charging and discharging. The active cores in the yolk-shell structure are generally movable and vulnerable, making it difficult to achieve robust electronic connections and protective effect with the outer conductive shells, as a result, leading that a thicker SEI film is coagulated on the YS-Fe_3O_4@C electrode.

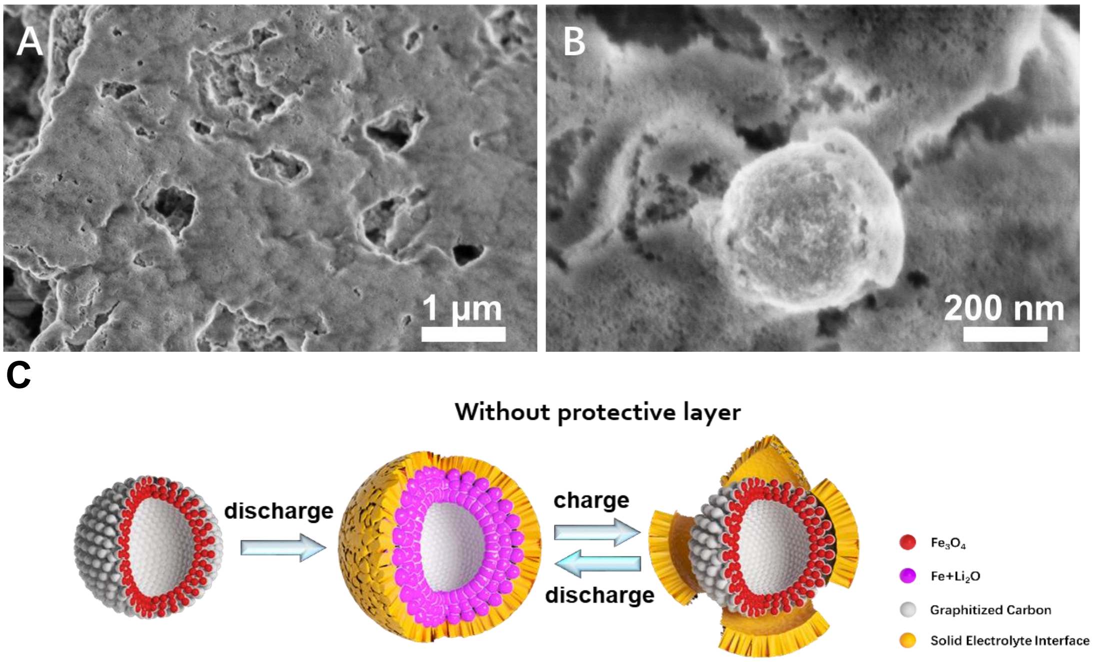

Supplementary Figure 29. Morphology of the hollow hybrid Fe_3O_4/C (HH-Fe_3O_4/C) electrode after 1000 cycles under a current density of 10 A g^{-1}. (A) the SEM image of the hollow hybrid Fe_3O_4/C electrode surface; (B) SEM image of the typical hollow hybrid Fe_3O_4/C nanosphere on the electrode; (C) Schematic illustrations for the structural change of the hollow Fe_3O_4@C nanospheres during fast charging and discharging. The morphology of hollow hybrid Fe_3O_4/C (HH-Fe_3O_4/C) electrode is almost entirely damaged after 1000 cycles due to the spallation of SEI film caused by the drastic volume change of Fe_3O_4 during the fast charging and discharging.

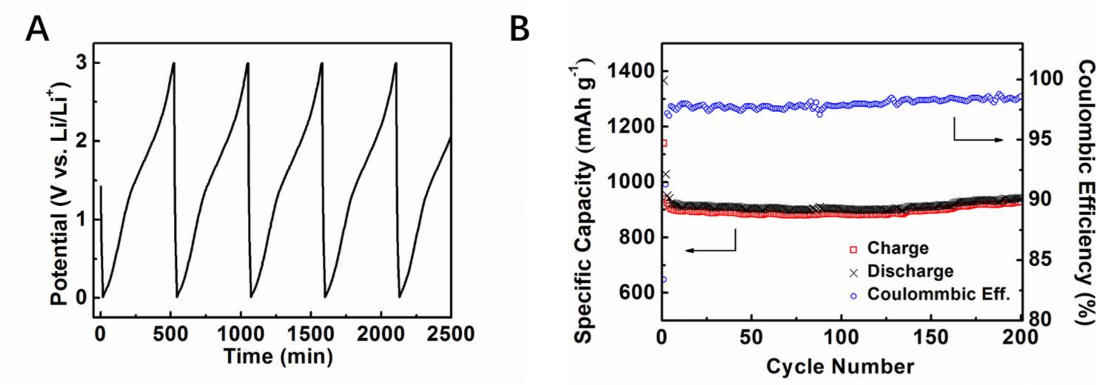

Supplementary Figure 30. Asymmetric charging and discharging performance of the hollow gradient-structured Fe_3O_4@C (HG-Fe_3O_4@C) electrodes. (A) Curves of charging at a charging current density of 0.2 A g^{-1} and discharging current density of 10 A g^{-1} in the voltage range from 0.05 to 3.00 V; (B) Cycling performance of the gradient-structured Fe_3O_4@C electrodes under the asymmetric charging and discharging condition.

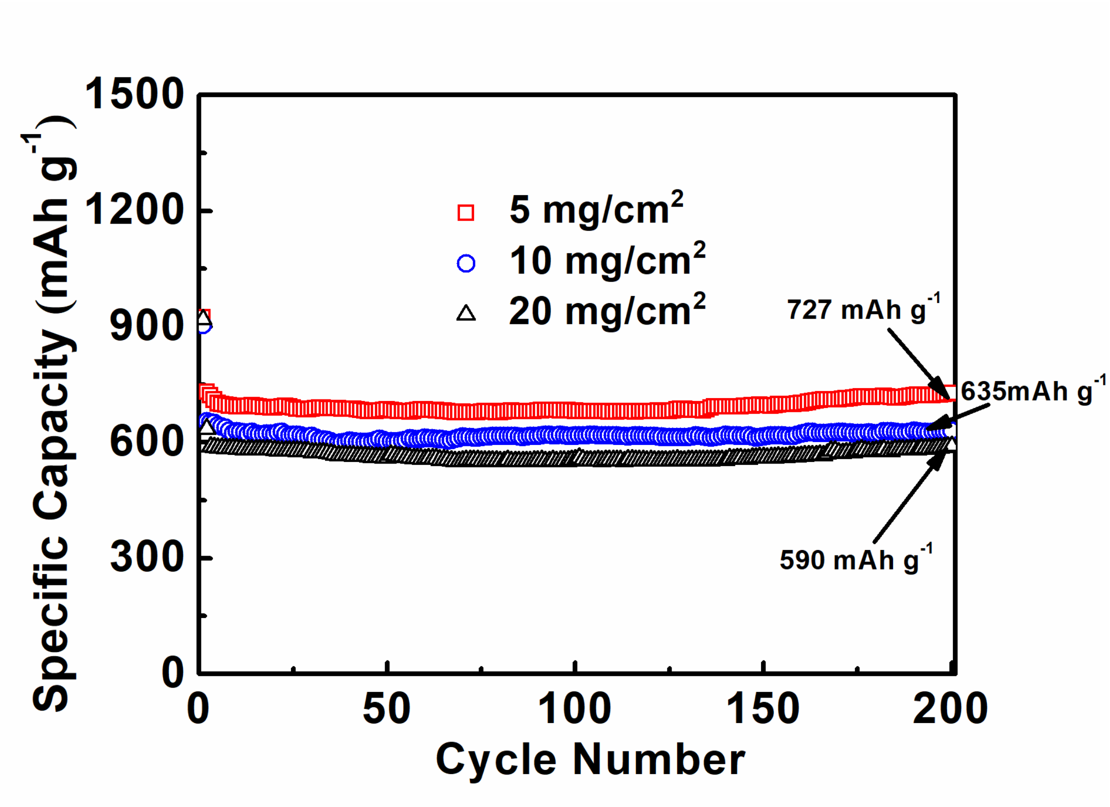

Supplementary Figure 31. The performance of the gradient-structured Fe_3O_4@C nanospheres anodes with high mass loading of 5, 10, and 20 mg/cm^2 at 10 A g^{-1}.

Supplementary Table 1. Comparison of the gradient-structured Fe_3O_4@C nanospheres with previous works.

Materials	Current densities/mA g^{-1}	Reversible specific capacities/mA h g^{-1}	Cycle life	Ref.
Gradient-structured Fe_3O_4@C nanospheres	10000	757	10000	This work
3D Porous $LiFeO_{2-x}$	100	1056	200	S1
Hierarchically Open-Porous Fe_3O_4	1000	798	300	S2
Three-Dimensional Graphene Foam Supported Fe_3O_4	890	785	500	S3
Hierarchical Fe_3O_4@Polypyrrole Nanocages	2000	650	500	S4

SWNT Anchored Fe_3O_4	90	850	200	S5
FeF_2			50	S6
Composite of $Fe_3O_4/MnCO_3$	100	617	100	S7
Fe_3O_4@Ti_3C_2 MXene hybrids	~5000	278.3	800	S8
Hierarchical Fe_3O_4@NC composites	200	450	2000	S9
Polypyrrole-coated Fe_2O_3@C composites	200	813		S10

Supplementary References

1. Y. Yang, X. Qu, X. Zhang, Y. Liu, J. Hu, J. Chen, M. Gao, and H. Pan, Higher Than 90% Initial Coulombic Efficiency with Staghorn-Coral-Like 3D Porous

$LiFeO_{2-x}$ as Anode Materials for Li-Ion Batteries, *Adv. Mater.* **32**, 1908285 (2020).

2. S. Yun, S. Bak, S. Kim, J. Yeon, M. Kim, X. Yang, P. Braun, Rational Design of Hierarchically Open-Porous Spherical Hybrid Architectures for Lithium-Ion Batteries. *Adv. Energy Mater.* **9**, 1802816 (2019).
3. J. Luo, J. Liu, Z. Zeng, C. Ng, L. Ma, H. Zhang, J. Lin, Z. Shen, and H. Fan, Three-Dimensional Graphene Foam Supported Fe_3O_4 Lithium Battery Anodes with Long Cycle Life and High Rate Capability. *Nano Lett.* **13**, 6136–6143 (2013).
4. J. Liu, X. Xu, R. Hu, L. Yang, M. Zhu, Uniform Hierarchical Fe_3O_4@Polypyrrole Nanocages for Superior Lithium Ion Battery Anodes. *Adv. Energy Mater.* **6**, 1600256 (2016).
5. Y. Kwon, K. Minnici, J. Park, S. R. Lee, G. Zhang, E. Takeuchi, K. Takeuchi, A. Marschilok, and E. Reichmanis, SWNT Anchored with Carboxylated Polythiophene “Links” on High-Capacity Li-Ion Battery Anode Materials. *J. Am. Chem. Soc.* **140**, 5666-5669 (2018).
6. K. Karki, L. Wu, Y. Ma, M. Armstrong, J. Holmes, S. Garofalini, Y. Zhu, E. Stach, and F. Wan, Revisiting Conversion Reaction Mechanisms in Lithium Batteries: Lithiation Driven Topotactic Transformation in FeF_2. *J. Am. Chem. Soc.* 140, 17915-17922 (2018).
7. Z. Yang, J. Wang, S. Yao, D. Sua, S. Liu, X. Feng, Composite of $Fe_3O_4/MnCO_3$ as anodes for lithium-ion batteries. *J. Alloy. Compd.* **757**, 112-117 (2018).
8. Y. Wang, Y. Li, Z. Qiu, X. Wu, P. Zhou, T. Zhou, J. Zhao, Z. Miao, J. Zhou, and S. Zhuo, Fe_3O_4@Ti_3C_2 MXene hybrids with ultrahigh volumetric capacity as an anode

material for lithium-ion batteries. *J. Mater. Chem. A*, **6**, 11189-11197 (2018).

9. F. Jiang, Y. Liu, Q. Wang, and Y. Zhou, Hierarchical Fe_3O_4@NC composites: ultra-long cycle life anode materials for lithium ion batteries. *J. Mater. Sci.* **53**, 2127-2136 (2018).

10. F. Han, D. Li, W. C. Li, C. Lei, Q. Sun, A. H. Lu, Nanoengineered polypyrrole coated Fe_2O_3@**C** multifunctional composites with an improved cycle stability as lithium-ion anodes, *Adv. Funct. Mater.* **23**, 1692-1700 (2013).
