# Supplementary material for: Inorganic-organic competitive coating strategy derived uniform hollow gradient-structured ferroferric oxide-carbon nanospheres for ultra-fast and long-term lithium-ion battery
Source: Nat Commun. 2021 May 20;12:2973. doi: 10.1038/s41467-021-23150-8 (PMC8137936; doi:10.1038/s41467-021-23150-8)
Supplement: Supplementary file 3 — Description of Additional Supplementary Files [file 41467_2021_23150_MOESM3_ESM.docx]

**Description of Additional Supplementary Files**

File Name: Supplementary Movie 1

Description: In-situ TEM characterization of the gradient-structured Fe_3_O_4_@C nanosphere based on the spinning projection technology. Scale bar: 100nm.

File Name: Supplementary Movie 2

Description: In-situ TEM characterization of the gradient-structured Fe_3_O_4_@C nanosphere based on the electron tomography technology. Scale bar: 100nm.
